# DFT Prediction of Structural and Physical Properties of Cr_3_AlC_2_ Under Pressure

**DOI:** 10.3390/nano15141082

**Published:** 2025-07-11

**Authors:** Jianhui Yang, Shenghai Fan, Haijun Hou, Qiang Fan

**Affiliations:** 1 College of Physics and Optoelectronic Engineering, Leshan Normal University, Leshan 614004, China; yangjianhui@lsnu.edu.cn; 2 Leshan West Silicon Materials Photovoltaic New Energy Industry Technology Research Institute, Leshan 614004, China; 3 School of Materials Engineering, Yancheng Institute of Technology, Yancheng 224051, Chinahhj@ycit.cn (H.H.); 4 School of New Energy Materials and Chemistry, Leshan Normal University, Leshan 614004, China

**Keywords:** Cr_3_AlC_2_, mechanical properties, electronic properties, thermal properties

## Abstract

This work explores the physical properties of the MAX-phase material Cr_3_AlC_2_ through the application of density functional theory (DFT). The refined lattice parameters were determined through the minimization of the total energy. In order to explore the electronic properties and bonding features, we carried out computations on the band structure and charge density distribution. The calculated elastic constants (*C*_ij_) validated the mechanical stability of Cr_3_AlC_2_. To assess the material’s ductility or brittleness, we calculated Pugh’s ratio, Poisson’s ratio, and Cauchy pressure. The hardness was determined. This study examined the anisotropic behavior of Cr_3_AlC_2_ using directional analyses of its elastic properties and by computing relevant anisotropy indicators. We examined several key properties of Cr_3_AlC_2_, including the Grüneisen parameter, acoustic characteristics, Debye temperature, thermal conductivity, melting point, heat capacity, Helmholtz free energy, entropy, and internal energy. Phonon dispersion spectra were analyzed to assess the dynamic stability of Cr_3_AlC_2_.

## 1. Introduction

Cr_3_AlC_2_, which belongs to the renowned MAX-phase family, exhibits unique attributes that combine the advantages of both metals and ceramics. The material is distinguished by its reduced density, superior resistance to oxidation, notable ductility, efficient electrical conductivity, self-lubricating properties, and favorable machinability [1,2,3,4,5]. These exceptional characteristics make Ti_3_AlC_2_ a material of significant technological interest, with great potential for applications at high temperatures. As a result, both Ti_3_AlC_2_ and its structurally analogous compound Ti_3_SiC_2_ have been extensively investigated [6]. These results are emphasized in a recent review article [7], focusing on layered, electrically conductive Ti_2_AlC and Ti_3_AlC_2_, as well as in another study by Barsoum [8]. Much of the prior research has focused on comprehending and modeling M_2_AX materials [9,10,11,12]. For M_3_AX_2_ phases, only a limited number of compounds have been examined, with Ti_3_SiC_2_ and Ti_3_AlC_2_ being notable exceptions [13]. However, as mentioned earlier, many M_3_AX_2_ compounds have recently been synthesized. For example, Ta_3_AlC_2_ was synthesized from metallic melts [14], whereas Ti_3_SnC_2_ was discovered by Dubois and colleagues [15]. Thus, a theoretical study of M_3_AX_2_ phases is both pertinent and essential for gaining insights into their characteristics. To date, it is commonly acknowledged that Ti_3_AlC_2_ and Ti_2_AlC exhibit superior oxidation resistance compared to Ti_3_SiC_2_ [16,17]. This exceptional oxidation resistance is believed to result from the development of a protective, dense Al_2_O_3_ surface layer. Given the assumption that other M_3_AlC_2_ compounds might also exhibit oxidation resistance similarly to Ti_3_AlC_2_, studying them would prove beneficial. To the best of our knowledge, apart from the significant case of Ti_3_AlC_2_, the properties of M_3_AlC_2_ compounds have not been thoroughly explored. The purpose of this study is to fill this research gap. The overall characteristics of the crystal structure, electronic properties, and elastic behavior of Cr_3_AlC_2_ were thoroughly examined.

## 2. Theoretical Methods

The characteristics of Cr_3_AlC_2_ were investigated by employing the DFT method, utilizing the Vienna ab initio simulation code [18]. The electron–nucleus interaction was modeled using pseudopotentials created through the projector-augmented wave (PAW) approach [19]. The exchange–correlation energy was calculated based on the generalized gradient approximation (GGA) in conjunction with the Perdew–Burke–Ernzerhof (PBE) functional [20]. Convergence tests indicated that a cutoff energy of 480 eV and *k*-point mesh of 8 × 8 × 10 were appropriate for achieving accurate results. An energy convergence criterion of 10^−4^ eV/cell was used, together with a force convergence threshold of 0.001 eV/Å for minimizing Hellmann–Feynman forces. Furthermore, the dynamic stability of Cr_3_AlC_2_ was assessed through phonon frequency analysis. Phonon computations were performed utilizing a 2 × 1 × 2 supercell, employing the Phonopy package [21,22].

## 3. Results and Discussion

### 3.1. Structural Properties

**Figure 1:**
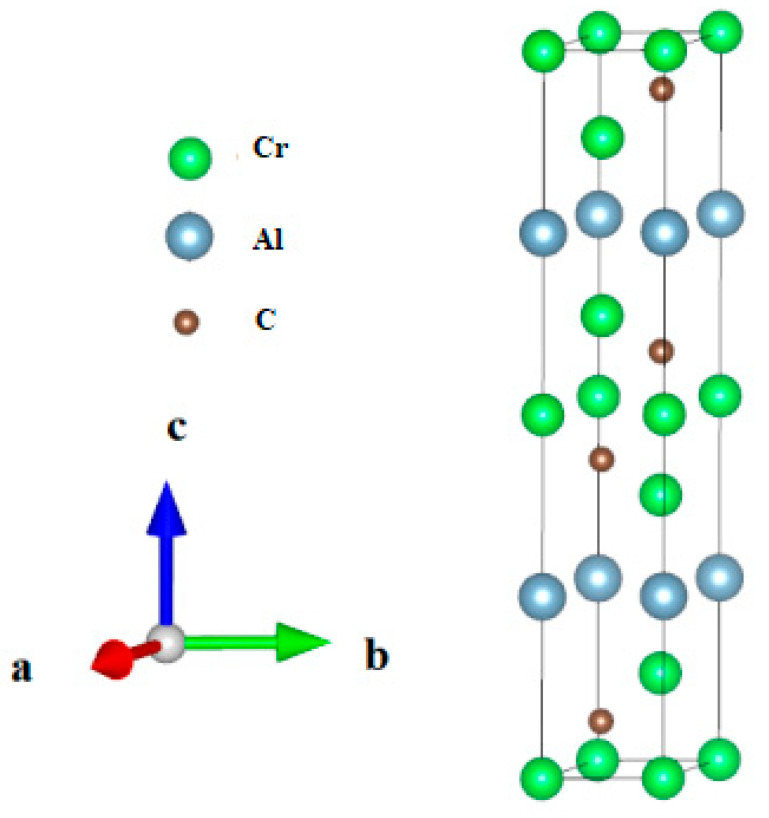
The crystal model of Cr_3_AlC_2_.

As shown in Figure 1, Cr_3_AlC_2_ adopts a hexagonal crystal structure belonging to the P63/mmc (194) space group. Table 1 presents the calculation results of this study as well as the relevant reference data [23]. The resulting values show reasonable consistency when contrasted with theoretical predictions. Notably, the difference between the computed lattice parameters and the theoretical values remains below 0.69%. Furthermore, Table 1 also provides the pressure-dependent lattice parameters, density, and volume.

### 3.2. Electronic Properties

**Figure 2:**
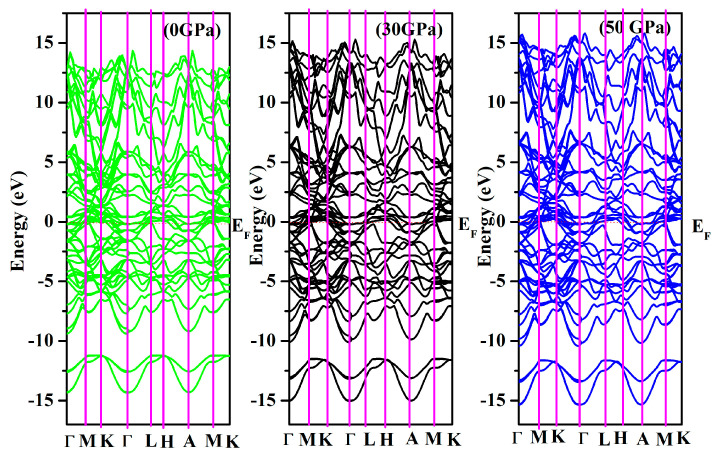
The band structure of Cr_3_AlC_2_ under various pressures.

**Figure 3:**
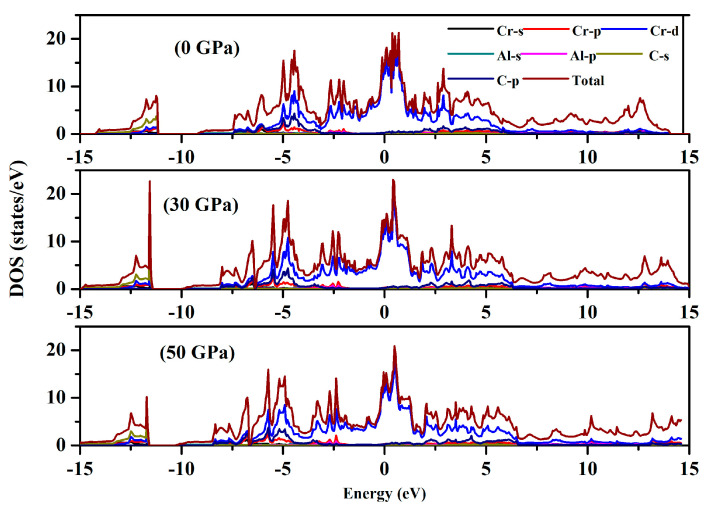
Density of states of Cr_3_AlC_2_ under various pressures.

In Figure 2, the band structure of Cr_3_AlC_2_ is presented. At E_F_ (Fermi energy level), the conduction and valence bands overlap, confirming the metallic nature of Cr_3_AlC_2_. Additionally, we calculated the density of states for Cr_3_AlC_2_ to analyze the bonding properties and contributions of various electronic states. The valence band is divided into two distinct sub-bands. (i) The lowest valence band spans from −15.0 to −10 eV for Cr_3_AlC_2_, where most of the states in this total density of states region are attributed to C-s orbitals, with minor contributions from Cr-s and Cr-p states. (ii) The upper valence band of Cr_3_AlC_2_ lies between −10 and 0.0 eV. It should be emphasized that the hybridization peak between Cr-d and C-p states appears in the lower-energy region, whereas the Cr-d peak is situated in the higher-energy range, close to the Fermi level (E_F_) (Figure 3).

**Figure 4:**
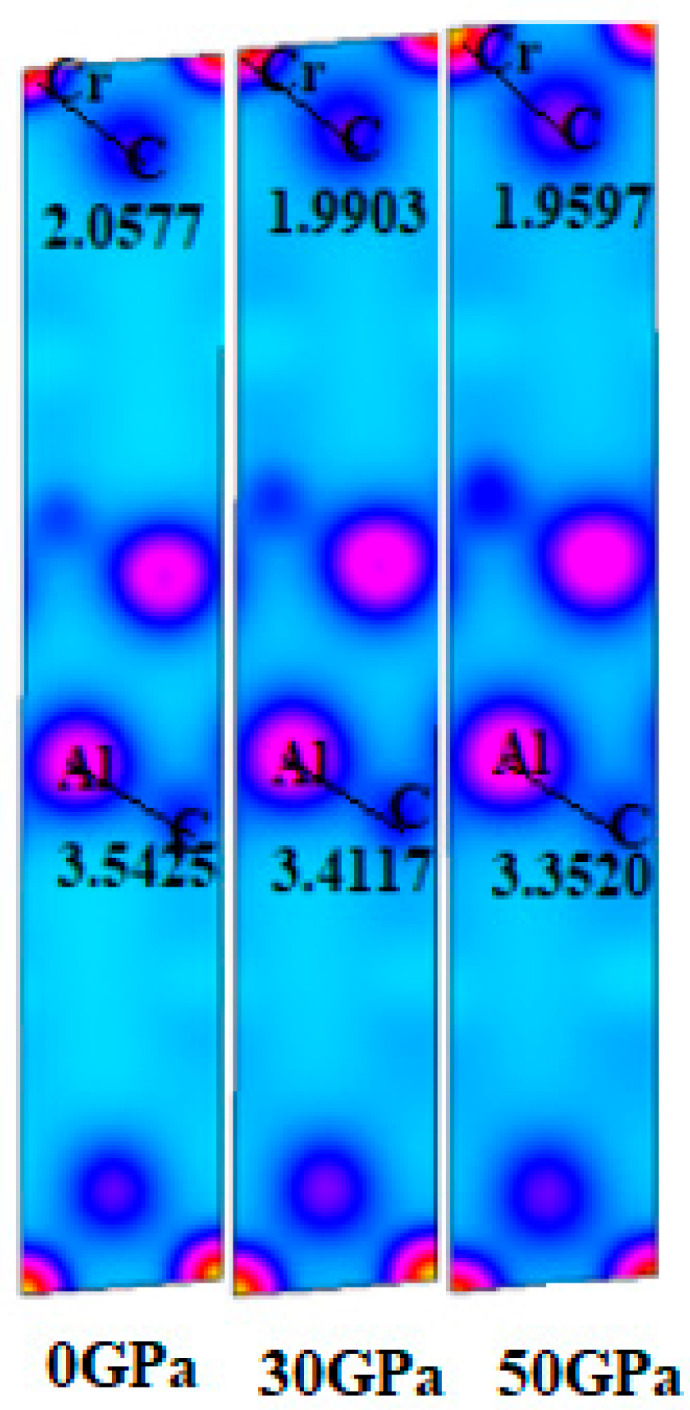
CDM of Cr_3_AlC_2_ under various pressures.

Figure 4 displays the charge density mapping (CDM) on the (101) plane, which provides insights into the bonding interactions among different atoms. With increasing pressure from 0 to 50 GPa, a significant enhancement in charge density is evident at specific locations. This elevated charge density enhances the bonding between Cr and C atoms, attributed to the overlapping of their electronic states. The CDM results indicate that the Cr-C bond exhibits greater strength compared to the Al-C bond.

### 3.3. Mechanical Properties

The elastic constants *C*_ij_ of Cr_3_AlC_2_ under various pressure conditions are presented in Table 2. Based on the Born criteria, the mechanical stability requirements at equilibrium are influenced by different crystal symmetries [24]:

*C*_44_ > 0; *C*_11_ + *C*_12_ − 2*C*_13_^2^/*C*_33_ > 0; and *C*_11_ − *C*_12_ > 0 (1)

As shown in Table 2, the calculated *C*_ij_ values for Cr_3_AlC_2_ align well with the corresponding theoretical values [23], suggesting that the current computational results are accurate. Based on the Voigt [25] and Reuss [26] methods, we calculated the values of bulk modulus (*B*) and shear modulus (*G*) using Hill’s approximation [27]. The computed values are summarized in Table 2, together with other previously reported results [23]. As shown in Table 2, the calculated *B*, *G*, and Young’s modulus (*E*) of Cr_3_AlC_2_ at 0 GPa are 207.0 GPa, 118.6 GPa, and 298.8 GPa, respectively, which align well with earlier findings. Table 3 presents Poisson’s ratio (*v*), *G*/*B* ratio, *C*_13_–*C*_44_, and *C*_12_–*C*_66_ values for Cr_3_AlC_2_ [28]. The mechanical behavior of brittleness or ductility can be evaluated using these parameters. A material is considered brittle if *ν* < 0.33, *G*/*B* > 0.57, *C*_13_–*C*_44_ < 0, and *C*_12_–*C*_66_ < 0; otherwise, it is classified as ductile. In Table 3, it is clear that Cr_3_AlC_2_ exhibits Poisson’s ratios (*ν*) below 0.33, *G*/*B* < 0.57, negative *C*_13_–*C*_44_ values, and negative *C*_12_-*C*_66_ values. These results indicate that Cr_3_AlC_2_ demonstrates brittle characteristics.

According to the literature [29,30,31], we can calculate various anisotropic parameters of the material such as three shear anisotropic factors, *A*_1_, *A*_2_, and *A*_3_, percentage anisotropy in compressibility (*A*_B_) and shear (*A*_G_), and the universal anisotropic index *A*^U^. As shown in Table 4, the Cr_3_AlC_2_ under consideration exhibits anisotropic behavior since the values of *A*_1_, *A*_2_, and *A*_3_ differ from unity. The non-zero values of *A*_B_, *A*_G_, and *A*^U^ (as shown in Table 4) confirm the anisotropic nature of Cr_3_AlC_2_.

**Figure 5:**
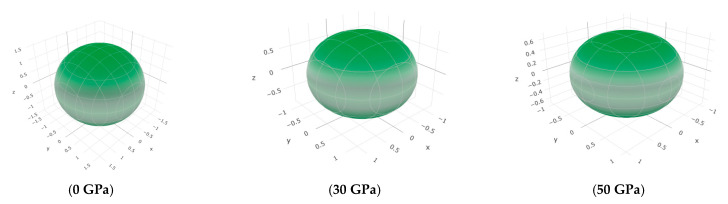
The *β* (TPa^−1^) surface characteristics of Cr_3_AlC_2_ under various pressures.

**Figure 6:**
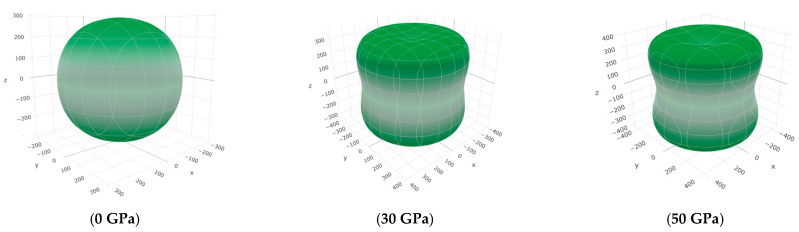
The *E* (GPa) surface characteristics of Cr_3_AlC_2_ under various pressures.

**Figure 7:**
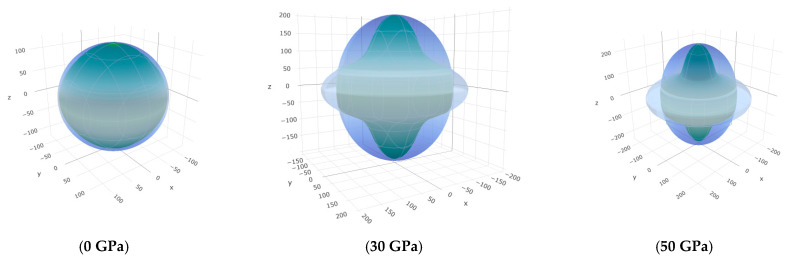
The *G* (GPa) surface characteristics of Cr_3_AlC_2_ under various pressures.

**Figure 8:**
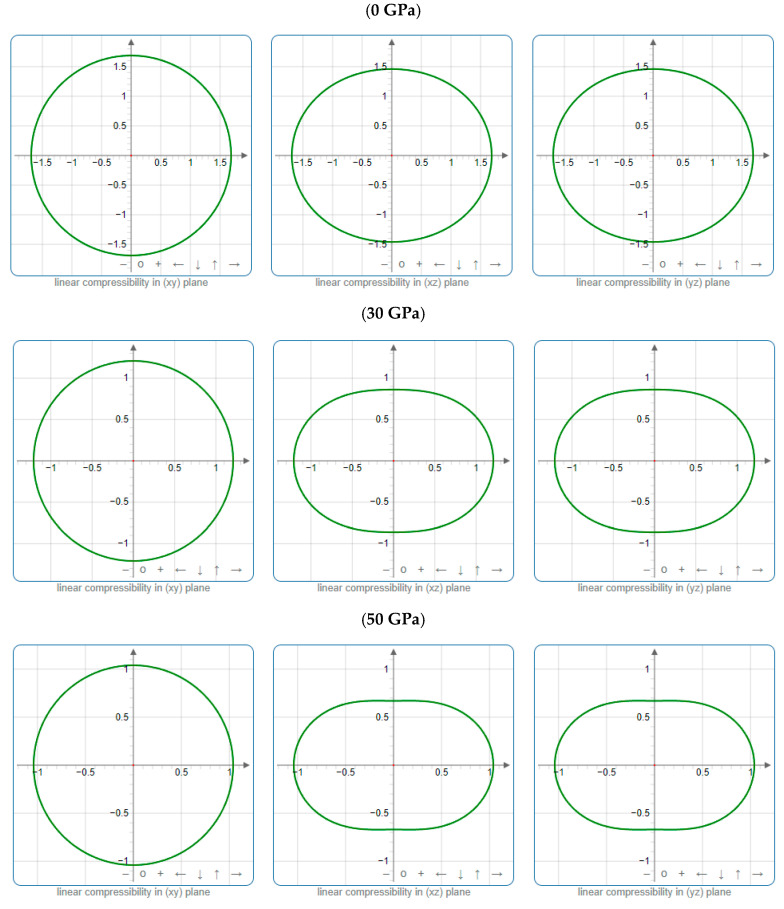
Projections of *β* (TPa^−1^) in different planes of Cr_3_AlC_2_ under various pressures.

**Figure 9:**
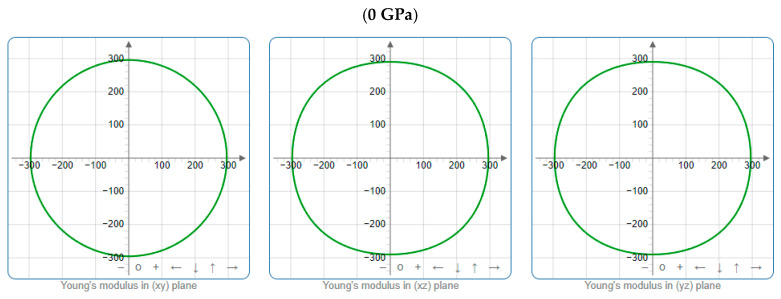

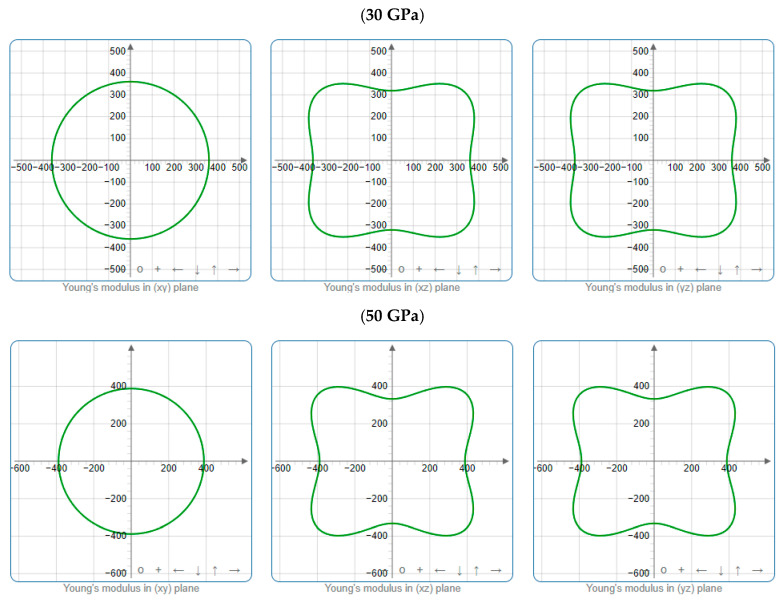
Projections of *E* (GPa) in different planes of Cr_3_AlC_2_ under various pressures.

**Figure 10:**
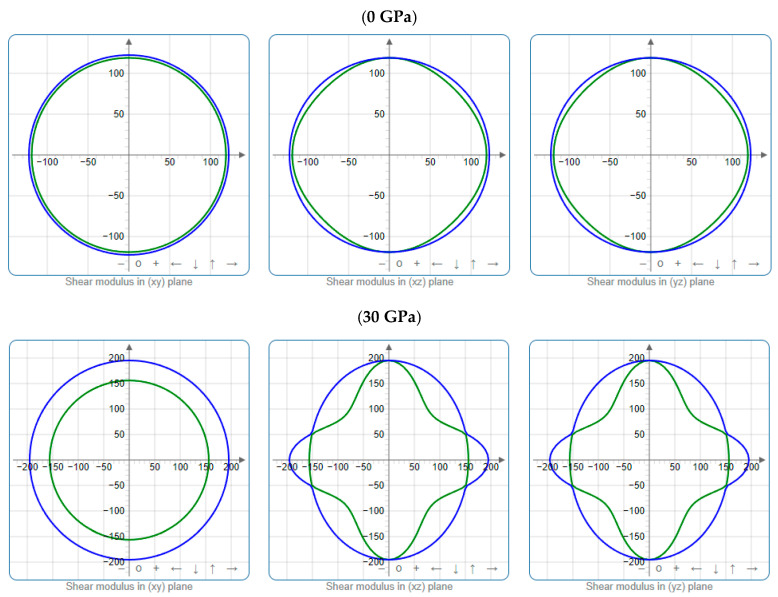

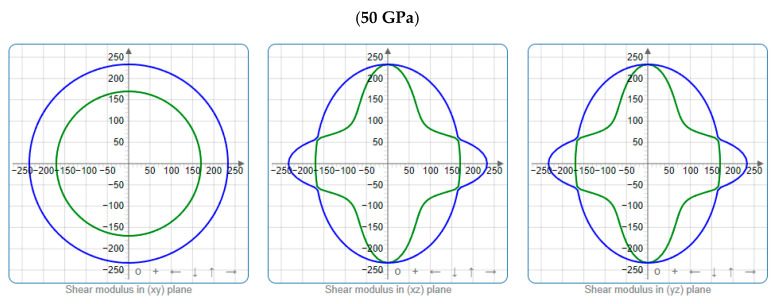
Projections of *G* (GPa) in different planes of Cr_3_AlC_2_ under various pressures.

To offer more distinct and thorough insight into the anisotropic properties of the investigated materials, we employed both two-dimensional (2D) and three-dimensional (3D) graphical representations of linear compressibility *β*, *E*, and *G* by ELATE software [32,33,34]. Figure 5, Figure 6, Figure 7, Figure 8, Figure 9 and Figure 10 present the 2D and 3D visualizations produced. A perfect sphere represents complete isotropy in solids, while deviations from this spherical shape indicate varying degrees of anisotropy (Figure 5, Figure 6 and Figure 7). Figure 8 illustrates the anisotropy in *β*, where the xy-plane shows isotropic behavior, and the xz- and yz-planes show anisotropic behavior. *E* also demonstrates anisotropic behavior, as depicted in Figure 9. This figure reveals that *E* exhibits isotropic properties within the xy-plane but displays anisotropic characteristics in the xz- and yz-planes. The graphs indicate that for Cr_3_AlC_2_, the blue line achieves its peak values along the axes in both the xz- and yz-planes, while it reaches its lowest value at a 45-degree angle relative to these axes. The shear modulus presents two surfaces in both 2D and 3D representations, as shown in Figure 10. Additionally, Table 5 provides the *β*_max_/*β*_min,_ *G*_max_/*G*_min,_ and *E*_max_/*E*_min_ values of *β*, *E*, and *G* for Cr_3_AlC_2_, confirming its significant anisotropic properties. As the pressure increases, the anisotropy also strengthens.

In this study, we computed both the Chen hardness (*H*_chen_) and the Miao hardness (*H*_miao_) [35,36].

(2) $$H_{Chen}=2{\left[{\left(\frac{G}{B}\right)}^{2}G\right]}^{0.585}-3$$

(3) $$H_{Miao}=\frac{(1-2v)E}{6(1+v)}$$

Fine’s principle establishes the criteria for defining the melting point of a solid [37,38].

*T*_m_ = 354 + 4.5(2*C*_11_ + *C*_33_)/3 (4)

The thermal conductivity *κ* of the material is obtained from the Clarke model [39]:

(5) $$\kappa_{\min}=0.87k_{B}Ma^{-\frac{2}{3}}\rho^{\frac{1}{6}}E^{\frac{1}{2}}$$

Here, *k*_B_ is Boltzmann’s constant, *ρ* is the density, *M*_a_ is the average mass per atom, and Young’s modulus (*E*).

Furthermore, based on a method in the literature, we were able to calculate the average sound velocity *v*_m_, the longitudinal velocity (*v*_l_), the transverse velocity (*v*_t_), and the Debye temperature *θ* [40]. In addition, the Grüneisen *γ*_a_ can be described by *v*_l_ and *v*_t_ [41]:

(6) $$\gamma_{a}=\frac{3}{2}(\frac{3v_{l}^{2}-4v_{t}^{2}}{v_{l}^{2}+2v_{t}^{2}})$$

The changes in *v*_l_, *v*_t_, *v*_m_, and *θ* under various pressures are summarized in Table 6. The *θ*, *v*_l_, *v*_t_, and *v*_m_ at 0 GPa are determined to be 702.4 K, 7.1182 km/s, 3.8357 km/s, and 4.2820 km/s, respectively. The values for *H*_V_, *T*_m_, γ_a_, and *κ* of Cr_3_AlC_2_ are provided in Table 6. The melting point of Cr_3_AlC_2_ is recorded as 1985.9 K under 0 GPa. If the maximum operational temperature is estimated as 0.8 *T*_m_, then the maximum service temperature of Cr_3_AlC_2_ approaches approximately 1300 °C, suggesting that Cr_3_AlC_2_ possesses favorable thermal stability for practical applications. As shown in Table 6, when the pressure increases, *H*_V_, *T*_m_, γ_a_, and *k* tend to rise. Furthermore, the hardness values for Cr_3_AlC_2_ obtained using Chen’s and Miao’s models at 0 GPa are 11.189 and 15.455 GPa, respectively. And our hardness is slightly greater than that of materials of the same type (Ti_3_AlC_2_ and Ti_3_SiC_2_) [13]. The hardness values calculated using the two methods are presented in Table 6. It has been observed that there is strong concurrence between them, suggesting the accuracy of the computation.

### 3.4. Dynamical Properties

**Figure 11:**
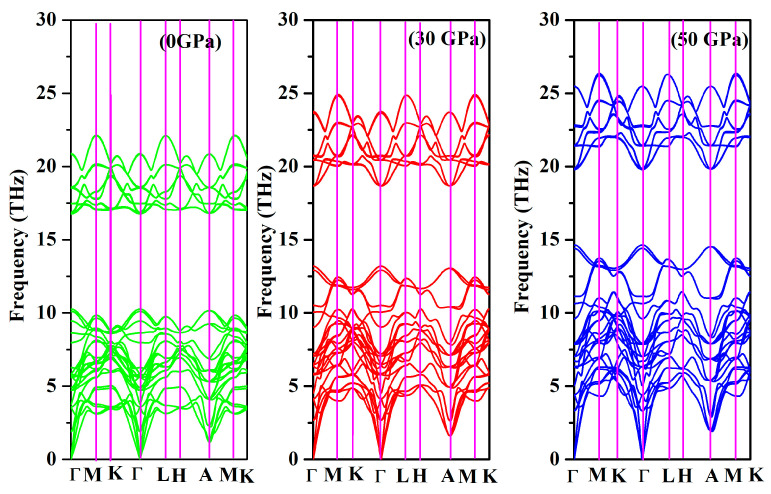
Phonon dispersion of Cr_3_AlC_2_ under various pressures.

**Figure 12:**
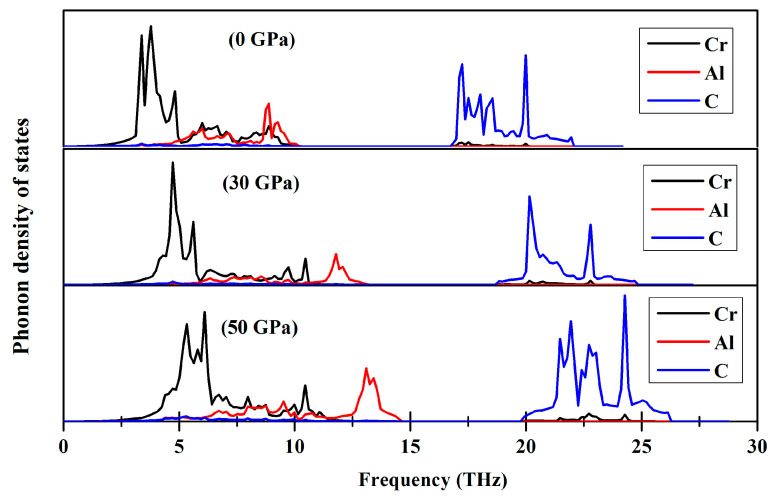
Phonon density of states of Cr_3_AlC_2_ under various pressures.

To ensure that the dynamical stability is thoroughly examined, it becomes imperative to conduct an analysis of the phonon dispersion of Cr_3_AlC_2_, when subjected to elevated pressure. The phonon dispersion and phonon density of state for Cr_3_AlC_2_ were calculated at 0 GPa, 30 GPa, and 50 GPa, as depicted in Figure 11 and Figure 12. It is clear that Cr_3_AlC_2_ exhibits dynamical stability, as no imaginary phonon frequencies are detected in this crystal structure. In correlation with the representation of the phonon density of state illustrated in Figure 12, the frequency range of 0–15 THz primarily corresponds to Cr and Al vibrations, while the frequency range of 15–25 THz predominantly corresponds to C vibrations.

### 3.5. Thermal Properties

**Figure 13:**
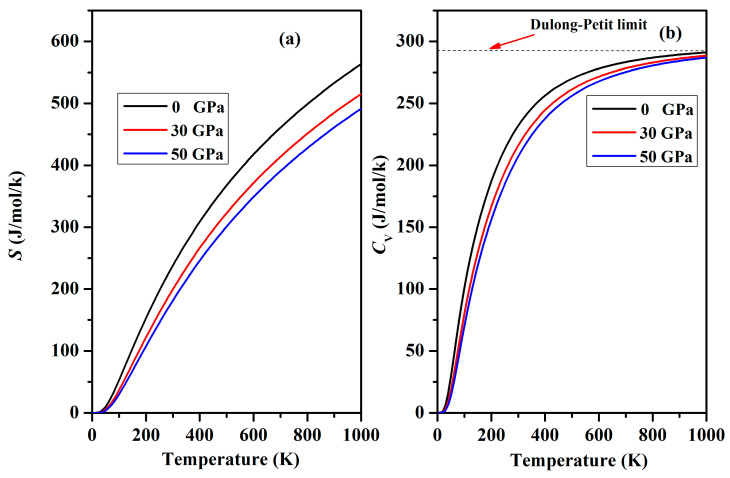
Changes in heat capacity *C*_V_ and entropy *S* with temperature under various pressures. (**a**) Heat capacity *C*_V_ (**b**) Entropy *S*.

The thermodynamic potential functions of Cr_3_AlC_2_ were calculated based on the phonon density of states obtained using the quasi-harmonic approximation [42]. These functions include the internal energy *E*, Helmholtz free energy *F*, and entropy *S*. Figure 13 shows *S* and *C*_V_ as functions of temperature. Below 50 K, the *S* values are nearly zero, as shown in Figure 13a. Above 100 K, there is a noticeable nonlinear increase, a trend that is quite common and becomes increasingly evident with increasing temperature as natural processes evolve. Figure 13b depicts *C*_V_ for Cr_3_AlC_2_ across the temperature interval from 0 to 1000 K. At *T* < 300 K, *C*_V_ follows the Debye *T*^3^ power law, while at *T* > 800 K, it closely aligns with the Dulong–Petit model. Table 7 presents *F* and *E* at various temperatures and pressures. These thermodynamic results are expected to provide a valuable reference for the practical utilization of Cr_3_AlC_2_.

## 4. Conclusions

In this study, a thorough investigation of Cr_3_AlC_2_ was conducted based on DFT. The mechanical properties were examined in detail under varying pressure conditions. Additionally, the calculated phonon dispersion further validates the material’s dynamical stability. This compound demonstrates a reasonable level of mechanical anisotropy alongside sufficient hardness. All thermal properties exhibit distinctive behavior across the pressure range from 0 to 50 GPa. However, these properties (*θ*, *γ*_a_, *T*_m_, and *k*) gradually increase as pressure rises to 50 GPa. We believe that these results will encourage researchers to delve deeper into this material, both through theoretical modeling and experimental exploration.

## Figures and Tables

**Table 1:** The optimized lattice parameters *a* and *c* (Å), density *ρ* (g/cm^3^), and volume *V* (Å^3^) of Cr_3_AlC_2_.

Pressure (GPa)		*a*	*c*	*ρ*	*V*
0	Present	2.8699	17.3922	5.5415	124.052
0	Ref. [23]	2.8508	17.272	5.66	121.56
10	Present	2.8260	17.1720	5.7881	118.767
20	Present	2.7896	16.9888	5.9269	115.986
30	Present	2.7572	16.8486	6.0464	113.693
40	Present	2.7287	16.7252	6.1557	111.694
50	Present	2.7040	16.6027	6.2567	109.872

**Table 2:** Calculated *C*_ij_ (GPa), elastic moduli (GPa), and *E* (GPa) of Cr_3_AlC_2_.

	Pressure (GPa)	*C*_11_	*C*_12_	*C*_13_	*C*_33_	*C*_44_	*C*_66_	*B*_V_	*B*_R_	*B*	*G*_V_	*G*_R_	*G*	*E*
Present	0	359.5	114.8	136.8	368.9	119.0	122.4	207.2	206.9	207.0	118.7	118.5	118.6	298.8
Theo. [23]	0	381	100	136	381	118	141			210			127	317
Present	10	408.9	134.9	169.4	415.9	148.9	137.0	242.3	241.8	242.1	137.6	136.4	137.0	345.8
Present	20	446.3	152	205.3	460.2	173.6	147.2	275.3	273.9	274.6	151.6	148.1	149.8	380.3
Present	30	484.7	172.7	238.4	491.7	195.3	156.0	306.7	305.0	305.8	163.4	157.0	160.2	409.2
Present	40	524.5	195.5	270.7	528.5	214.8	164.5	339.0	337.0	338.0	174.9	165.8	170.3	437.5
Present	50	554.4	214.9	299.0	565.0	233.2	169.7	366.6	363.8	365.2	184.6	172.7	178.6	460.8

**Table 3:** Calculated *G*/*B*, Poisson’s ratio *v*, *C*_13_–*C*_44_, and *C*_12_–*C*_66_ of Cr_3_AlC_2_.

Pressure (GPa)		*G/B*	*v*	*C*_13_–*C*_44_	*C*_12_–*C*_66_
0	Present	0.573	0.2595	17.8	−7.6
0	Theo. [23]	0.61	0.25		
10	Present	0.566	0.2619	20.5	−2.1
20	Present	0.546	0.2692	31.7	4.8
30	Present	0.524	0.2770	43.1	16.7
40	Present	0.504	0.2843	55.9	31
50	Present	0.493	0.2897	65.8	45.2

**Table 4:** Calculated elastic anisotropic factors of Cr_3_AlC_2_.

Pressure (GPa)	*A* ^U^	*A*_B_ (%)	*A*_G_ (%)	*A*_1_	*A*_2_	*A*_3_
0	0.0064	0.0006	0.0001	1.0466	1.0466	1
10	0.0508	0.0044	0.0034	1.2255	1.2255	1
20	0.1206	0.0111	0.0040	1.4003	1.4003	1
30	0.2135	0.0203	0.0031	1.5637	1.5637	1
40	0.2817	0.0271	0.0017	1.6794	1.6794	1
50	0.3588	0.0338	0.0046	1.7890	1.7890	1

**Table 5:** The values of *β*_max_/*β*_min,_ *G*_max_/*G*_min,_ and *E*_max_/*E*_min_ of Cr_3_AlC_2_ under various pressures.

Pressure (GPa)	*β*_max_/*β*_min_	*G*_max_/*G*_min_	*E*_max_/*E*_min_
0	1.1565	1.08	1.042
10	1.2024	1.234	1.175
20	1.3580	1.421	1.302
30	1.4025	1.599	1.44
40	1.4434	1.727	1.536
50	1.5528	1.847	1.607

**Table 6:** The hardness (*H*_Chen_ and *H*_Miao_) (GPa), wave velocity (*v*_l_, *v*_t_, *v*_m_) (km/s), Debye temperature *θ* (K), Grüneisen constant *γ*_a_, melting point *T*_m_ (K), and thermal conductivity *k* (W.m^−1^ K^−1^) of Cr_3_AlC_2_.

Pressure (GPa)	*H*_Chen_	*H*_Miao_	*v*_l_	*v*_t_	*v*_m_	*θ*	*γ*_a_	*T*_m_	*k*
0	14.03	19.02	8.1173	4.6262	5.1417	702.4	1.5465	1985.9	1.8586
Ti_3_AlC_2_ 0 [13]	9.2								
Ti_3_SiC_2_ 0 [13]	11.1								
10	15.27	21.75	8.5666	4.8651	5.4088	749.7	1.5591	2204.6	2.0140
20	15.44	23.05	8.9460	5.0274	5.5941	781.6	1.5966	2383.2	2.1205
30	15.29	23.82	9.2684	5.1473	5.7330	806.3	1.6386	2545.7	2.2069
40	15.11	24.49	9.5810	5.2598	5.8635	829.6	1.6794	2720.3	2.2888
50	14.99	25.05	9.8199	5.3428	5.9601	847.9	1.7109	2864.7	2.3553

**Table 7:** The calculated *F* (KJ/mol) and *E* (KJ/mol) of Cr_3_AlC_2_ under various pressures.

Temperature/K	0 GPa		30 GPa		50 GPa	
	*F*	*E*	*F*	*E*	*F*	*E*
300	43.31	114.81	62.43	122.38	72.42	126.92
400	15.85	139.34	39.03	145.54	50.96	149.30
500	−18.04	165.71	9.51	170.89	23.55	174.06
600	−57.36	193.14	−25.26	197.57	−9.00	200.30
700	−101.32	221.23	−64.57	225.09	−46.02	227.47
800	−149.34	249.75	−107.86	253.17	−86.99	255.28
900	−200.95	278.57	−154.69	281.63	−131.46	283.53
1000	−255.79	307.61	−204.71	310.38	−179.10	312.09

## Data Availability

Data are contained within the article.
